# In Vitro impairment of whole blood coagulation and platelet function by hypertonic saline hydroxyethyl starch

**DOI:** 10.1186/1757-7241-19-12

**Published:** 2011-02-10

**Authors:** Alexander A Hanke, Stephanie Maschler, Herbert Schöchl, Felix Flöricke, Klaus Görlinger, Klaus Zanger, Peter Kienbaum

**Affiliations:** 1 Department of Anaesthesiology and Intensive Care Medicine, Hannover Medical School, Germany; 2 Department of Anaesthesiology, University Hospital Düsseldorf, Germany; 3 Department of Anaesthesiology and Intensive Care, AUVA Trauma Hospital, Salzburg, Austria; 4 Department of Anaesthesiology and Intensive Care Medicine, University Hospital Essen, Germany; 5 Institute for Anatomy II, University Hospital Düsseldorf, Germany

## Abstract

**Background:**

Hypertonic saline hydroxyethyl starch (HH) has been recommended for first line treatment of hemorrhagic shock. Its effects on coagulation are unclear. We studied in vitro effects of HH dilution on whole blood coagulation and platelet function. Furthermore 7.2% hypertonic saline, 6% hydroxyethylstarch (as ingredients of HH), and 0.9% saline solution (as control) were tested in comparable dilutions to estimate specific component effects of HH on coagulation.

**Methods:**

The study was designed as experimental non-randomized comparative in vitro study. Following institutional review board approval and informed consent blood samples were taken from 10 healthy volunteers and diluted in vitro with either HH (HyperHaes^®^, Fresenius Kabi, Germany), hypertonic saline (HT, 7.2% NaCl), hydroxyethylstarch (HS, HAES6%, Fresenius Kabi, Germany) or NaCl 0.9% (ISO) in a proportion of 5%, 10%, 20% and 40%. Coagulation was studied in whole blood by rotation thrombelastometry (ROTEM) after thromboplastin activation without (ExTEM) and with inhibition of thrombocyte function by cytochalasin D (FibTEM), the latter was performed to determine fibrin polymerisation alone. Values are expressed as maximal clot firmness (MCF, [mm]) and clotting time (CT, [s]). Platelet aggregation was determined by impedance aggregrometry (Multiplate) after activation with thrombin receptor-activating peptide 6 (TRAP) and quantified by the area under the aggregation curve (AUC [aggregation units (AU)/min]). Scanning electron microscopy was performed to evaluate HyperHaes induced cell shape changes of thrombocytes.

Statistics: 2-way ANOVA for repeated measurements, Bonferroni post hoc test, p < 0.01.

**Results:**

Dilution impaired whole blood coagulation and thrombocyte aggregation in all dilutions in a dose dependent fashion. In contrast to dilution with ISO and HS, respectively, dilution with HH as well as HT almost abolished coagulation (MCF_ExTEM _from 57.3 ± 4.9 mm (native) to 1.7 ± 2.2 mm (HH 40% dilution; p < 0.0001) and to 6.6 ± 3.4 mm (HT 40% dilution; p < 0.0001) and thrombocyte aggregation (AUC from 1067 ± 234 AU/mm (native) to 14.5 ± 12.5 AU/mm (HH 40% dilution; p < 0.0001) and to 20.4 ± 10.4 AU/min (HT 40% dilution; p < 0.0001) without differences between HH and HT (MCF: p = 0.452; AUC: p = 0.449).

**Conclusions:**

HH impairs platelet function during in vitro dilution already at 5% dilution. Impairment of whole blood coagulation is significant after 10% dilution or more. This effect can be pinpointed to the platelet function impairing hypertonic saline component and to a lesser extend to fibrin polymerization inhibition by the colloid component or dilution effects.

Accordingly, repeated administration and overdosage should be avoided.

## Background

Normovolemia and sufficient coagulation capacity are major goals during early resuscitation of traumatized patients with hemorrhagic shock. Nevertheless, significant morbidity and mortality are related to coagulopathy due to loss and consumption of coagulation factors as well as volume substitution induced hemodilution. After patient admission to the emergency care department definite strategies have been established to improve outcome after severe hemorrhagic shock [1] including transfusion of packed red blood cell concentrates, fresh frozen plasma, cryoprecipitate and coagulation factor concentrates. However, during the prehospital period various crystalloids and colloids have been suggested for treatment of hemorrhagic shock. Whatever fluid is administered, there is at least a dose dependent dilution of coagulation factors which is associated with a further impairment of coagulation.

Recently, small volume resuscitation by intravenous administration of small amounts of hypertonic saline hydroxyethyl starch has been introduced for rapid restoration of normovolemia following severe trauma. However, both hypertonic sodium chloride as well as hydroxyethyl starch, impair coagulation and platelet function; the former by altering plasma clotting times and platelet aggregation [2], the latter by decreasing FVIII plasma concentration and by interference with fibrin polymerization and thus decreasing clot strength [3-6]. Nevertheless, in a porcine model of hemorrhagic shock and resuscitation, in general, the least effects on coagulation were observed following small volume resuscitation by administration of hypertonic saline hydroxyethyl starch for resuscitation [7]. Since small volume resuscitation was associated with alterations in the coagulation system in this animal model as well, we evaluated these complex effects on coagulation and thrombocyte function in vitro in human whole blood and tested the hypothesis that HyperHaes causes impaired whole blood coagulation and platelet function.

## Methods

The study was designed as experimental non-randomized comparative in vitro study.

Following institutional review board approval (study number: 2953, University Hospital Düsseldorf) this study was conducted in accordance with the Helsinki Declarations and European Unions Convention on Human Rights and Biomedicine.

The guidelines for reporting non-randomized studies [8] were utilized in the drafting of this report.

### Blood samples

Ten volunteers (six male/four female; average age 33.7 years (range: 26-42 y)) of Caucasian origin participated in the study after oral and written information and written consent. All volunteers were healthy and free of medication. Blood was taken from a basilic vein using an 18-gauge IV catheter and collected in both citrated and heparinzed tubes (Vacutainer, Becton Dickenson, Heidelberg, Germany).

### Sample preparation

Blood was assigned to four different groups: Group A (HH): Hypertonic Saline Hydroxyethyl Starch (HyperHaes^®^, Fresenius Kabi, Bad Homburg, Germany); Group B (HT): 7.2% hypertonic sodium chloride solution; Group C (HS): 6% hydroxyethyl starch (HAES 200/0.5 6%, Fresenius Kabi, Bad Homburg, Germany); Group D (ISO): isotonic sodium chloride solution, serving as control group.

Blood samples were diluted with one of the four fluids (HH, HT, HS and ISO) in a fix proportion of 1:20 (5% dilution), 1:10 (10% dilution), 1:5 (20% dilution), 1:2.5 (40% dilution) and the effects of dilution were compared to undiluted baseline values.

### Whole blood coagulation

Whole blood coagulation was analyzed by rotation thrombelastometry (ROTEM, TEM international, Munich, Germany) in citrated whole blood samples. The technique has been described previously elsewhere [9-11]. In brief, ROTEM analyzes viscoelastic clot characteristics over time in activated whole blood and recognizes both the time course of clotting as well as the firmness of the resulting clot. The following commercially available tests were performed following the manufacturer's instructions: ExTEM (extrinsically activation by tissue factor) and FibTEM (extrinsically activation by tissue factor with addition of Cytochalasin D to inhibit platelet function and display fibrin polymerization only - all tests Pentapharm, Munich, Germany). Since maximum clot firmness (MCF) in whole blood coagulation is mainly determined by platelet function and fibrin polymerization, while clotting times (CT) are dependent on the speed of thrombin generation by clotting factors [10] the chosen parameters were: CT quantifying the time from beginning of the reaction until start of clot formation and MCF indicating clot stability at its highest degree.

Since samples for thrombelastometry are recommended to be analysed within two hours we used three ROTEM devices in parallel. Tests were performed in a standard sequence. ROTEM devices were chosen in a random order.

### Platelet function

Platelet function was determined by multiple electrode aggregometry (MEA) using a novel multiple platelet function analyzer (Multiplate, Dynabyte, Munich, Germany, heparinized whole blood samples) following TRAP activation (thrombin activating peptide, TRAP-test, Dynabyte, Munich, Germany). The technique has been described previously elsewhere [11]. MEA utilizes single uses test cells. These cells contain two pairs of sensor wires extending into a 50% diluted whole blood sample. Platelets are non adhaesive in resting state, but when activated stick to the sensor wires enhancing electrical impedance between wires. These impedance changes are recorded over a period of six minutes. Tests were performed regarding the manufacturer's instructions. As indicator for platelet function the area under the aggregation curve (AUC) was determined indicating overall platelet activity.

### Electron microscopy

Scanning electron microscopy (SEM) was performed at 1:2000 and 1:5400 magnification on samples to evaluate effects of HyperHaes on the cell shape of the thrombocytes, using a Jeol 35 CF SEM and documentation by Orion 6.60 software (Orion Microscopy, Belgium).

### Statistical analysis

A power analysis was performed based on results of a previously performed pilot test. Assuming an alpha error of 0.05 with a power of 0.95 we calculated a necessary sample size of 8 to show a significant effect of a 10% dilution of HH on MCF in the EXTEM test. Based on this calculation and to ensure reasonable data we have chosen to increase sample size to 10.

After positive testing on normal distribution (Shapiro-Wilk-test) two way ANOVAs with Bonferroni post-hoc testing were performed for statistical analysis. The Statistical Package for Social Sciences (SPSS for Windows, 13.0, SPSS Inc., Chicago, IL., USA) and GraphPad Prism (Version 4.02, GraphPad Software Inc., San Diego, CA., USA) were used.

Values are displayed median ± standard deviation. Considering a confidence interval of 99% an α-error below 0.01 was considered to be statistically significant.

## Results

### Whole blood coagulation

**Figure 1:**
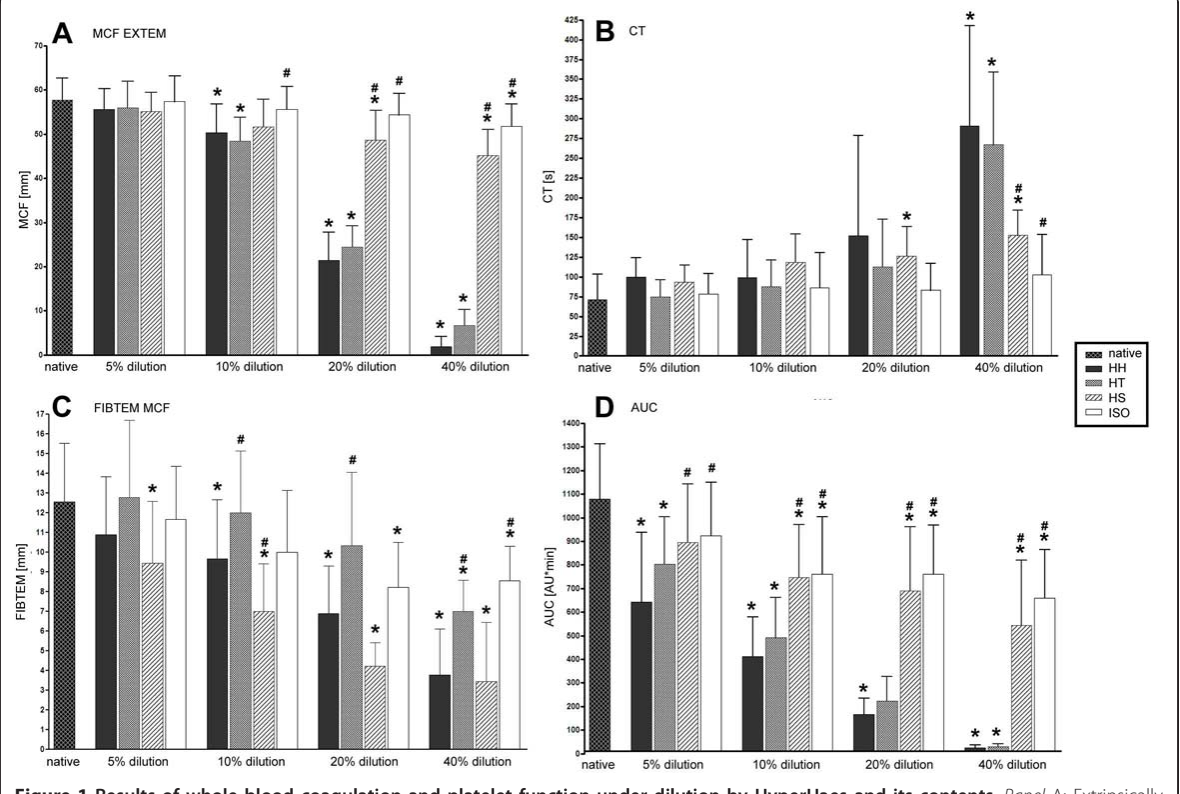
**Results of whole blood coagulation and platelet function under dilution by HyperHaes and its contents**. *Panel *A: Extrinsically activated measurements of maximum clot firmness (ExTEM-MCF in millimeter) and Panel B: coagulation time after extrinsically activation (CT in seconds). Panel C: Maximum clot firmness of fibrin polymerization (FibTEM-MCF in millimeter) and Panel D: AUC of platelet aggregation after thrombin activation (AUC in aggregation units (AU)*millimeter). Measurements were performed with respect to dilution of 5%, 10%, 20%, and 40%. Tested groups are HyperHaes (HH), hypertonic saline solution (HT), Haes 6% (HS) and isotonic saline solution (ISO). * are assigned with significantly different results as compared to baseline values. # are assigned with significantly different results as compared to HH results. Note that HH and HT treatment lead to comparable impairment of ExTEM-MCF and AUC indicating HT to be responsible for HH's impairment of platelet function and whole blood coagulation.

Maximum clot firmness (MCF) in rotational thrombelastometry after extrinsically activation (ExTEM) showed a dose dependent impairment in all tested groups (figure 1A). In the control group ISO significant differences to baseline were found at 40% dilution (p = 0.0001). In HH and HT significant influence on MCF was found when dilution was ≥ 10% (HH: p = 0.0009; HT: p = 0.0002). HS impaired MCF statistically significant when dilution was ≥20% (p = 0.0033). No differences were found between HH and HT (p = 0.452). HS (p < 0.0001) and ISO (p < 0.0001) showed less impairment of MCF compared to HH.

Clotting times (CT) were statistically significant prolonged in all tested groups but the control group ISO (figure 1B). ISO did not induce significant differences as compared to baseline (ISO 40% dilution; p = 0.128). Significant influence on CT was found in HH and HT when dilution was 40% (HH: p = 0.0003; HT: p = 0.0002). HS already impaired CT statistically significant when dilution was 20% (p = 0.0022).

Fibrin polymerization (FibTEM) was statistically significant impaired in all tested groups (figure 1C). In the control group (ISO) MCF as compared to baseline was significantly reduced when dilution was ≥20% (p = 0.0005). Significant reduction of MCF by HH was found when dilution was ≥10% (p < 0.0001). HT significantly impaired MCF at 40% dilution (p = 0.0006). MCF was significantly reduced by HS throughout the test beginning at 5% dilution (p = 0.0033).

### Platelet function

AUC was significantly impaired in all tested groups including ISO in a dose dependent fashion (figure 1D). As compared to baseline ISO and HS significantly decreased AUC when dilution was ≥10% (ISO: p = 0.0022; HS: p = 0.0002). AUC was significantly decreased in HH and HT in all tested dilutions beginning at 5% dilution (HH: p = 0.0001; HT: p = 0.0014). Between HH and HT no significant differences were found (p = 0.449) while impairment of platelet function in HH was pronounced compared to HS (p = 0.0011) and ISO (p < 0.0001).

### Electron microscopy

**Figure 2:**
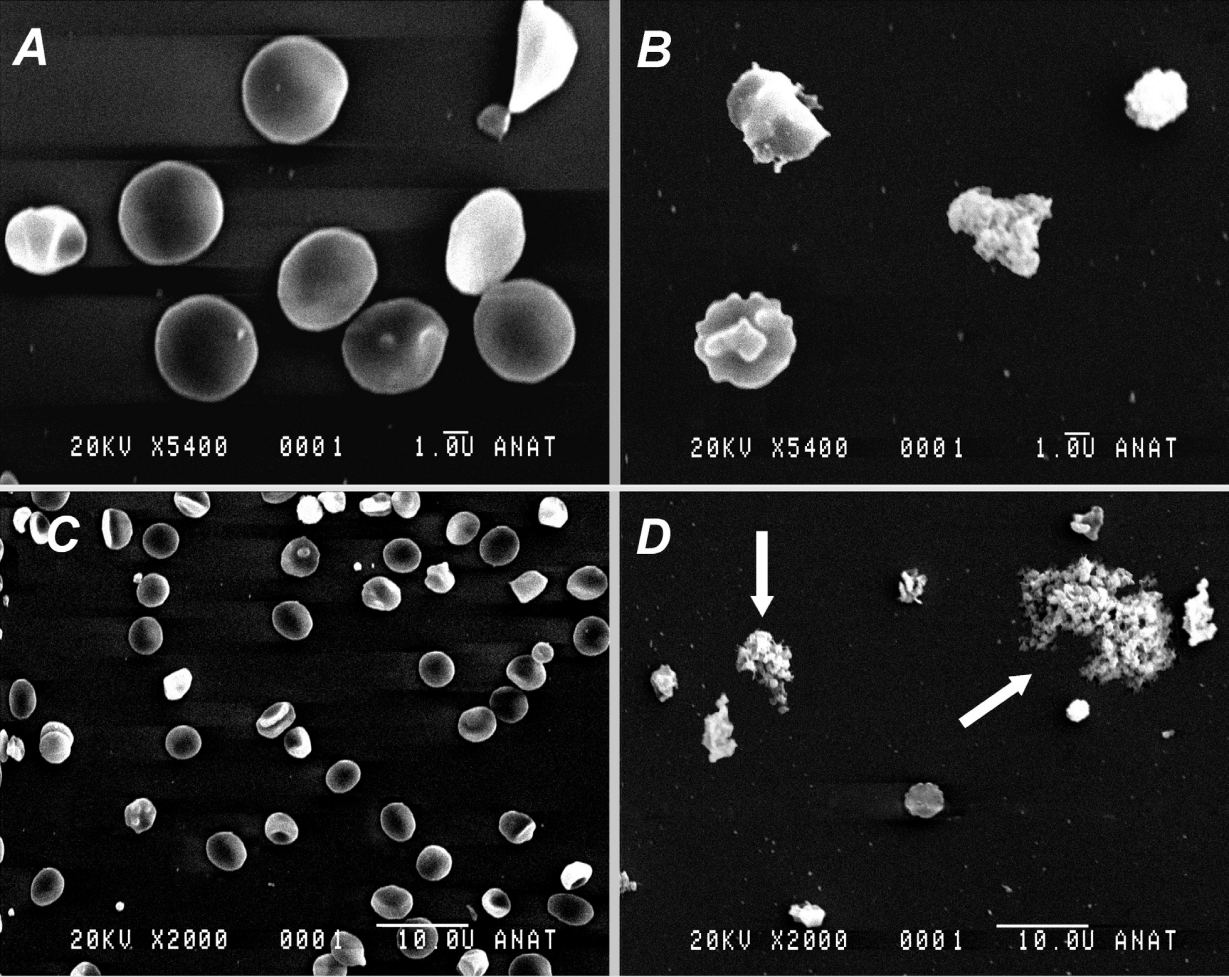
**Scanning electron microscopy of native platelets (panels A and C) and platelets from blood after 40% dilution HyperHaes (panels B and D) in 5400fold (panels A and B) and 2000fold (panels C and D) magnification**. Representative scans demonstrate deformed platelets, spreading activated platelets (panel B), as well as large aggregates of activated platelets (arrows in panel D). Note small bars on the lower right side of each panel indicating length of 1.0 U = 1 μm (panels A and B) and 10.0 U = 10 μm (panels C and D), respectively.

Dilution with HH caused deformed platelets and large aggregates of platelets (figure 2). Since building of aggregates prohibits exact counting of platelets within these aggregates a quantification of morphological changes was impossible.

## Discussion

HH significantly impairs whole blood coagulation and platelet function in a dose dependent fashion in vitro by reducing platelet function as well as fibrin polymerization. The mechanism can be attributed to the hypertonic saline component and is associated with a dehydration and activation of platelets leading to accumulation of thrombocytes as demonstrated by scanning electron microscopy.

HH is suggested for first line treatment in hemorrhagic shock. Since studies in trauma patients are always affected by an inhomogeneous cohort of patients we have chosen a model of in vitro dilution for standardization of study conditions to estimate the effects of HyperHaes and to identify a possible coagulation impairing substance. Since our study was not designed to evaluate effects on circulatory conditions, we did not adapt dilution volumes of the different agents to possible hemodynamic potentials but in a fixed manner as compared to HH infusion alone. Furthermore the study cannot assess or predict effects on blood loss or outcome.

In vitro studies on coagulation are limited because complex hemostasis pathways cannot be simulated in a complete natural way. Interaction between primary and secondary hemostasis cannot be displayed in coagulation tests. Regular laboratory tests on coagulation use plasma as matrix for analysis. Therefore we decided to use rotational thrombelastometry and multiple platelet aggregation which assay whole blood as a more physiologically matrix to assess coagulation including platelet function. Furthermore thrombelastometry analyzes the end product of coagulation: the clot itself and its stability over time, which indicates clot building potential at the time of analysis. A dynamic time course of coagulation impairment and possible recovery from impairment cannot displayed in our study.

In vivo osmolarity is influenced by numerous factors. Osmolarity in dogs after a 50% blood volume withdrawal and following infusion of 4 ml*kg^-1 ^hypertonic NaCl (2400 mOsmol*l^-1^, which is comparable to HyperHaes) led to an increase of plasma osmolarity from 307 mOsmol*l^-1 ^to 333 mOsmol*l^-1 ^within 30 minutes [11]. Estimating average plasma osmolarity of 300 mOsmol*l^-1 ^and an osmolarity of 2400 mOsmol*l^-1 ^for HyperHaes in vitro dilution by 5% would suggest a resulting osmolarity of approximately 405 mOsmol*l^-1 ^which is already markedly above physiological levels. These in vitro high osmolarity conditions could compromise the translation of the results into clinical settings. Nevertheless, it remains unclear if compensation mechanisms are able to adjust osmolarity before interfering with platelets. In a different setting of acidosis and diminished coagulation laboratory parameters did not return to normal after compensation of acidosis [12]. Furthermore it could be possible that repeated administration or overdosage of HH could account for a non-physiological increase in osmolarity exceeding possibilities of compensation.

Normal blood volume in adults may be estimated to be 70 - 80 ml/kg bodyweight. Accordingly, the recommended HH dose of 4 ml/kg bodyweight in patients with hemorrhagic shock yields a hemodilution of 1:17.5 (5.7%) to 1:20 (5%). Since this mirrors normal conditions without blood loss we have chosen a 5% dilution as lowest degree of dilution for our study. Blood loss would lead to a further reduction in circulating blood volume and thus to a relatively increased portion of infused HH per ml blood volume resulting in an increased test agent/blood ratio, *ergo *to greater dilution. Blood loss of 50% blood volume then would lead to approximately 1:10 (10%) dilution, 75% blood loss would account for a 1:5 (20%) dilution and 40% dilution would be comparable to 87.5% blood loss. With respect to this consideration increasing blood loss would lead to increasing relative overdosage accounting for possible enhancement of otherwise induced coagulation disorders.

Even 5% whole blood dilution with HH significantly impaired platelet function. This effect on thrombocytes cannot be adequately detected in whole blood coagulation. However, MCF was affected in all samples with ≥10% dilution and CT prolongation finally occurred when dilution was 40%. Maximum clot firmness in whole blood coagulation is basically determined by platelet function and fibrin polymerization, while clotting times are dependent on the speed of thrombin generation by clotting factors [13]. Thus, HH affected platelet function and fibrin polymerization in a more severe way than action of clotting factors. Responsible for interference with fibrin polymerization of HH is its HS portion, since we demonstrate a comparable impairment of fibrin clot firmness by HH as compared to HS. It is well known that HS inhibits fibrin polymerization [14-18]. Our data are consistent with these findings. This effect is most likely caused by dilution of fibrinogen [19] and decreased FXIIIa-mediated fibrin cross linking [14,15]. However, the precise molecular mechanism still remains unclear.

The mechanism of action of HH to improve blood pressure is based on mobilization of extravasal fluids along an osmotic gradient by intavasal administration of HH [20]. We suspected this intavasal hyperosmolarity also to be one possible mechanism of interaction between the hypertonic solution and platelets leading to dehydrated and functionless thrombocytes. Platelets treated with and without HH were examined by electron microscopy. In the HH dilution deformed single platelets as well as large aggregates of activated platelets can be seen (figure 2). Such aggregates could account for a loss of platelet function and in vivo could lead to an obstruction of small vessels leading to a reduced platelet count as well. A detection of such aggregates after in vivo administration of hypertonic saline solution has not been done to date and would be of great interest concerning our findings.

In experimental settings controversial effects of HH on coagulation have been described. In animal models of uncontrolled hemorrhage treatment with hypertonic saline led to an aggravation of hemorrhage [21-23]. In these studies only hypertonic saline was studied while HS was not administered alone or in combination with hypertonic saline. In a recent study in a model of uncontrolled hemorrhage in pigs after liver injury less hemorrhage after HH administration was observed as compared to the use of colloids alone [7]. However, in this study red blood cells collected by an automated cell saver were simultaneously to the test agent infused. As a consequence the dosage of the hypertonic and hyperoncotic agent was reduced in a relative way by the parallel infusion of red blood cells which could have weakened the coagulation impairing effect of HH. Despite this, to reflect comparable hemodynamic potential greater volumes of colloid infusions were admitted leading to a higher dilution of clotting factors in the control group. Since red blood cell concentrates or cell saver blood is available in the hospital only the settings of this study are more comparable to an admission in the emergency room or the operating theatre than to a preclinical situation. As a consequence conclusions on the influence of these solutions on coagulation and blood loss in a preclinical situation should be drawn with caution.

Another hazard might occur when hypertonic saline is used in combination with large doses of colloids due to additional risks of adverse effects of colloids itself as for example anaphylactic reactions or reduction of kidney function which also have to be considered [24-27].

In different clinical situations of major blood loss such as penetrating chest trauma [28], patients undergoing cardiac surgery [29,30], or vascular surgery [31-33] studies indicating beneficial effects on outcome have been published. However, results of meta-analysises showed if any only minor improvement of survival no matter if hypertonic saline solution is used exclusively or in combination with colloids [34-36].

Our results indicate HH to cause a dose dependent impairment of platelet function and whole blood coagulation. However, these effects appear to be small in dilutions comparable to expected dilution after treatment of shock when the circulating blood volume is not reduced. From a different point of view this implicates that considering a small therapeutic index the risk of overdosage seems to be high and should be strictly avoided. Whether this also accounts for repeated admission and length of a time interval for possible safe repeated administration of HH cannot be assessed in the present study and may be addressed in future investigations.

Furthermore, the recommended dosage of HH is calculated with respect to bodyweight. In clinical situations variables as for example body weight can be assessed easily. In preclinical situations it is much more difficult to assess the patient's bodyweight which could lead to overdosage *per se*.

We calculated our dilution series to compare resulting dilution effects to HH treatment at different degrees of severe blood loss. Since we found greater effects on platelets with increasing dilution due to higher drug levels, we suspect HH treatment to show increasing negative effects on coagulation and platelet function with increasing blood loss due to possible relative overdosage. HH is designed to help stabilizing circulatory conditions in these situations. This implicates that dosage in patients with higher blood loss should be calculated with care, repeated administration should be avoided and the physician should be aware of increasing coagulopathy.

Since it remains questionable if our findings can be transferred into clinical settings clinical studies are necessary to evaluate such issues.

## Conclusions

HyperHaes as an example for hypertonic saline hydroxyethyl starch solution impairs whole blood coagulation and platelet function in a dose dependent fashion. Responsible for impairment of platelet function is the hypertonic saline component, while interference with fibrin polymerization is based on both colloid and dilution effects.

Overdosage and relative overdosage due to underestimated blood loss should be avoided and increasing coagulopathy considered in a subtle manner.

## Competing interests

Dr. Hanke and Dr. Schöchl received speaker fees from CSL Behring, Marburg, Germany, Dr. Görlinger received speaker fees from CSL Behring, Marburg, Germany, and TEM international, Munich, Germany.

## Authors' contributions

AH conceived of the study, carried out the experiments, performed statistical analysis of the results and drafted the manuscript. SM performed essential laboratory work. HS, FF and KG participated in the design of the study and interpretation of the results. KZ performed electron microscopy. PK participated in study design and coordination and helped to draft the manuscript. All authors read and approved the final manuscript.
